# Development of Novel Superabsorbent Hybrid Hydrogels by E-Beam Crosslinking

**DOI:** 10.3390/gels7040189

**Published:** 2021-10-29

**Authors:** Ion Călina, Maria Demeter, Anca Scărișoreanu, Marin Micutz

**Affiliations:** 1 National Institute for Lasers Plasma and Radiation Physics, 409 Atomiștilor, 077125 Măgurele, Romania; calina.cosmin@inflpr.ro; 2 Department of Physical Chemistry, University of Bucharest, 4-12 Regina Elisabeta Blvd., 030018 Bucharest, Romania; micutz@gw-chimie.math.unibuc.ro

**Keywords:** hydrogel, superabsorbent, e-beam, swelling, crosslinking

## Abstract

In this study, several superabsorbent hybrid hydrogel compositions prepared from xanthan gum (XG)/sodium carboxymethylcellulose (CMC)/graphene oxide (GO) were synthesized by e-beam radiation crosslinking. We studied and evaluated the effects of GO content from the chemical structure of the hydrogels according to: sol-gel analysis, swelling degree, diffusion of water, ATR-FTIR spectroscopy, network structure, and dynamic mechanical analysis. The gel fraction and swelling properties of the prepared hydrogels depended on the polymer compositions and the absorbed dose. The hybrid XGCMCGO hydrogels showed superabsorbent capacity and reached equilibrium in less than 6 h. In particular, the XGCMCGO (70:30) hydrogel reached the highest swelling degree of about 6000%, at an irradiation dose of 15 kGy. The magnitude of the elastic (G′) and viscous (G″) moduli were strongly dependent on the absorbed dose. When the degree of crosslinking was higher, the G′ parameter was found to exceed 1000 Pa. In the case of the XGCMCGO (80:20) hydrogel compositions, the Mc and ξ parameters decreased with the absorbed dose, while crosslinking density increased, which demonstrated that we obtained a superabsorbent hydrogel with a permanent structure.

## 1. Introduction

In the specialized scientific literature, there are reports on a series of composite hydrogels that have various characteristic properties and can have many applications: delivering or controlled releasing of drugs [1,2,3], adsorbents for water purification [4,5,6], adsorbents of dyestuffs in various fields [7], and energy-storage devices [8,9]. Most hydrogels containing graphene oxide (GO) in their composition were obtained using chemical crosslinking agents.

Huang et al. developed some superabsorbent hydrogels based on different GO concentrations (GO/poly (acrylic acid-*co*-acrylamide)) by in situ radical solution polymerization, and obtained hydrogels with a swelling capacity of up to 1100% [10,11].

Hydrogel crosslinking using ionizing radiation is a well-established method that is clean and rapid, and that ensures the obtaining of sterile products with a permanent and homogeneous network structure.

By varying the absorbed dose, the degree of crosslinking, on which the swelling degree strongly depends, can be controlled. Since it has many hydrophilic groups on its surface, GO is an excellent material that can be used in mixtures with several polymers in order to obtain new hydrogels with superabsorbent properties [12,13,14]. Natural polymers such as CMC and XG are more susceptible to degradation when they are irradiated in aqueous solutions due to the indirect effect of water. The effect of e-beam irradiation on XG, which is used as an ingredient in the food industry to determine irradiation stability, has been investigated by Li et al. [15]. The study showed that the viscosity of irradiated aqueous XG-based solutions was reduced as the irradiation dose increased (5–50 kGy), suggesting degradation or depolymerizations of polysaccharide macromolecules.

Comprehensives studies on the γ-radiation of XG in solid and aqueous states were performed by Șen and Hayrabolulu et al. They showed that the effect of γ-radiation in air for XG is chain scission, which is effective when the dose rate is decreased due to the enhanced oxidative degradations during irradiation [16,17]. The same research group developed superabsorbent hydrogels based on XG using γ-radiation crosslinking synthesis at very low absorbed dose, such as 1–2 kGy [18].

Another study investigated how the irradiation dose (0–30 kGy) influenced the viscosity of CMC solutions, and developed methods to control the degradation [19].

Most often, the crosslinking by irradiation of natural polymers is accompanied by the degradation of the polymer chain. Despite this major disadvantage, natural polymers are preferred over the petroleum-derived synthetic polymers for ecological reasons, but also due to the fact that natural polymers have inherent properties of biocompatibility and biodegradability. Thus, materials produced for biomedical purposes, in addition to being easier to process, are much more easily accepted by cells and tissues.

One of the reasons for using graphene oxide (GO) to create hybrid hydrogels with natural polymers such as xanthan gum (XG) and caboxymethylcellulose (CMC) in their composition is primarily related to obtaining a hydrogel with improved mechanical properties compared to the initial polymers. It is well known that natural polymers have low mechanical properties, although they have excellent absorption properties due to their highly hydrophilic nature.

GO induces elasticity of polymer chains, thermal and chemical stability. It is a material suitable for increasing the mechanical strength of natural polymers and due to the ease with which GO can be dispersed in a polymer mixture increasing the miscibility of these compounds [20,21].

A particularly important role that GO can play in the composition of some hydrogels is related to the lyophilization process when the hydrogels lose their water content. GO hydrogels obviously maintain their chemical composition and still have high porosity, a large specific surface area, and a high affinity for various molecules. Recent studies have shown an increased drug-loading capacity and prolonged release when using GO-based hydrogel systems, making them suitable for advanced drug-delivery applications [22]. In our previous study, superabsorbent hydrogels incorporated with GO were obtained by e-beam irradiation in the absence of air, at low radiation doses (0.5–3 kGy), from a high concentration of polymers in aqueous solution (paste-like condition) [23]. The compositions of the hydrogels above were characterized by a lower swelling capacity and a higher crosslinking density.

The present study sought to develop and characterize a new hybrid hydrogel with superabsorbent properties composed exclusively of biodegradable polymers (XG and CMC), incorporated with GO, to obtain a hydrogel with the best structural, rheological, and swelling properties, and that could be used in the field of biomedical engineering [24] and in hygienic products.

## 2. Results and Discussion

### 2.1. Sol-Gel Analysis

**Figure 1:**
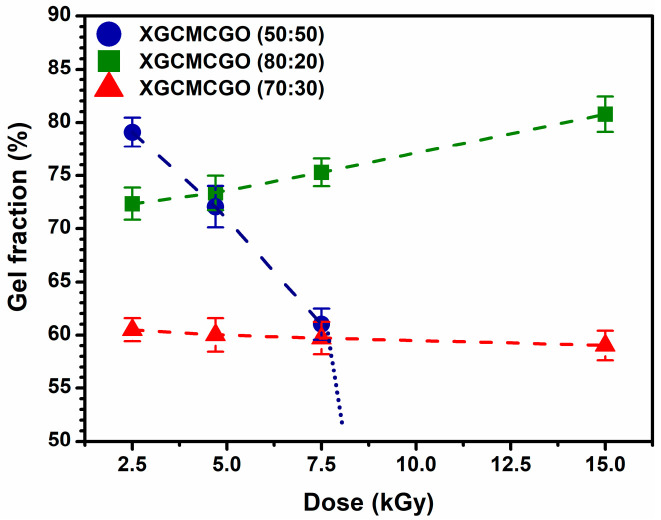
Gel fraction as a function of the absorbed dose during e-beam irradiation for the XGCMCGO hydrogels.

Figure 1 shows the variation of the gel fraction (GF) depending on the absorbed dose of the XGCMCGO hydrogels with compositions of 50:50, 80:20, and 70:30. For these hydrogel compositions, it was observed that the highest percentage of the GF was obtained at the lowest absorbed dose: 78% for XGCMCGO (50:50) and 73% for XGCMCGO (80:20); while for XGCMCGO (70:30), at the lowest dose, it obtained the lowest percentage of the GF, namely 60%. The XGCMCGO (70:30) hydrogel showed an almost constant value of GF over the entire range of applied radiation doses.

For the hydrogel samples having the compositions of 50:50 and 70:30, the percentage of GF decreased with an increasing absorbed dose. The exception to this rule was the hydrogel with equal concentrations of polymers (XGCMCGO (50:50)), for which the determination of the GF was no longer possible at doses above 7.5 kGy, due to the advanced degradation of the samples after irradiation.

For the XGCMCGO (80:20) hydrogel, the percentage of the GF increased with an increasing absorbed dose; the highest percentage of the GF was 78% at an irradiation dose of 15 kGy.

The obtained values were comparable to those of other studies regarding the e-beam crosslinking of similar hydrogel compositions. For example, Sung et al. prepared CMC/GO hydrogels by e-beam irradiation at 30 kGy and obtained a gel fraction of 67.3% [20].

Another study, performed by Said et al., which investigated the formation of CMC/AA hydrogels in aqueous solutions under the effect of e-beam irradiation, showed that the gel fraction increased quickly with a dose of 50 kGy, reaching a value of 70%, then in the dose range of 50–100 kGy, showed a slight increase [25].

The rate of hydrogel formation depends on the *p*_0_/*q*_0_ ratio of the polymer. If the crosslinking process predominates over the degradation process, an insoluble gel is formed as a detriment to the degradation process [26]. When polymers are subjected to ionizing radiation, crosslinking and main chain scission occur simultaneously. The quantitative estimation of the extent of crosslinking and degradation can be made using the Charlesby–Rosiak equation. The *p*_0_/*q*_0_ ratios calculated using the Charlesby–Rosiak equation for the XGCMCGO hydrogels are presented in Table 1.

The lowest value of the *p*_0_/*q*_0_ of 0.64 was obtained for the hydrogel composition of 80:20, which contained the highest XG concentration. The XGCMCGO (50:50) and XGCMCGO (70:30) hydrogels showed *p*_0_/*q*_0_ ratios of 1.04 and 0.66, respectively. It is well known in the radiation-chemistry field that when various polymeric composition subjected to a treatment with ionizing radiation show a *p*_0_/*q*_0_ less than 2, the crosslinking processes predominated [27]. In the present study, these values were slightly higher compared to those obtained for hydrogels prepared with high concentrations of acrylic acid (70% AA) in data presented in our previous study, where the *p*_0_/*q*_0_ ratio was equal to 0.28 [23].

The gelation dose (D_g_) is another parameter that shows the minimum dose required to obtain an insoluble gel during irradiation. D_g_ values decreased as the concentration of polymers and crosslinking agent (*N*′*N*—methylenebis(acrylamide), NMBA) increased. We observed that the gelation occurred at a very low dose (0.23 kGy) for the XGCMCGO (50:50) hydrogel.

The radiation-chemical yield of crosslinking (G(X)) decreased with an increase in the irradiation dose for all the XGCMCGO hydrogel compositions. The maximum value of this parameter was 132.2 µmol/J for the XGCMCGO (70:30) hydrogel at a dose of 2.5 kGy. For the radiation-chemical yield of scission (G(S)), the maximum value was 252.5 µmol/J for the XGCMCGO (50:50) hydrogel at a dose of 2.5 kGy (Table 2).

As shown in the results presented in Table 2, G(S) > G(X) for the XGCMCGO (50:50) hydrogel, with approximately double the value. Therefore, it was obvious that in this case, the degradation processes predominated.

On the other hand, we observed that G(X) > G(S) for the XGCMCGO (80:20) and XGCMCGO (70:30) hydrogels. In these cases, the crosslinking predominated.

### 2.2. Swelling Degree

To characterize the network structure and determine the effective crosslinking density of the XGCMCGO hydrogels, the swelling properties in deionized water (DI) were first investigated.

**Figure 2:**
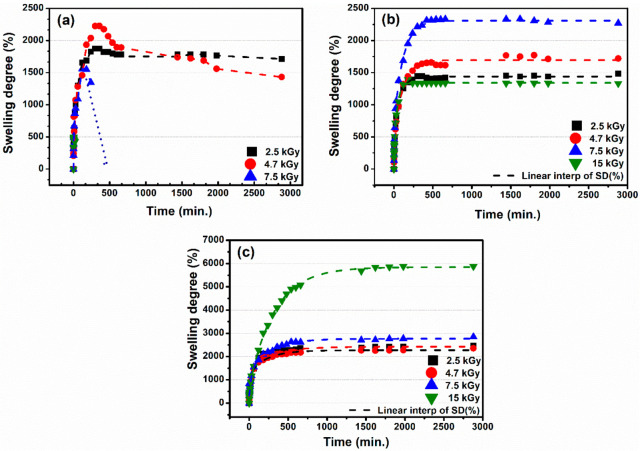
The swelling degree of the hydrogels in DI water: (**a**) XGCMCGO (50:50); (**b**) XGCMCGO (80:20); (**c**) XGCMCGO (70:30).

Figure 2 shows the variation of the swelling degree (SD) at equilibrium, as a function of time and the absorbed dose, for the XGCMCGO hydrogels. All hydrogels showed good swelling ability. The maximum SD obtained at a dose of 15 kGy was ~6000%, which classified these XGCMCGO (70:30) hydrogels as “superabsorbent hydrogels”. In the case of hydrogels with low concentrations of CMC, XG, AA, and NMBA, the SD had a value of 2250% for the XGCMCGO (50:50) hydrogel obtained at 4.7 kGy. At irradiation doses greater than 5 kGy, this hydrogel was degraded in deionized water (DI) in less than 2 h. For the XGCMCGO (80:20) hydrogel, the SD showed a maximum value of 2300% at a dose of 7.5 kGy.

Moreover, we can specify that these hydrogels had the ability to reach equilibrium in less than 6 h and were stable in deionized water; of these, the most suitable proved to be the XGCMCGO (70:30) hydrogel. Previous studies of superabsorbent hydrogels based on CMC/AA/montmorillonite obtained by γ-irradiation showed a swelling degree of 16,000% [28]. Compared to our study, the above study used a high concentration of polymers; i.e., 10% CMC and 30% AA. Sultana et al. synthesized copolymer hydrogels from acrylamide/CMC by γ-irradiation, and they obtained an SD value in DI water of 795% [29]. We have shown that by using low concentrations of polymers and GO and appropriate mass ratios between them, with a moderate radiation dose rate, stable hydrogels with different degrees of swelling were obtained.

Superabsorbent hydrogels based on xanthan gum/polyacrylic acid/graphene oxide were prepared as absorbers for removing methylene blue dye from the water. For these hydrogels, the maximum swelling value in the neutral medium was found to be 2100% for a GO concentration of 1% [30]. Superabsorbent resin based on acrylic acid/CMC/GO showed the highest swelling capacity with 0.6% of GO in hydrogel composition, about 750 g g^−1^ in distilled water. The above study demonstrated that the incorporation of a moderate amount of GO in a CMC-based hydrogel can improve the water capacity of the material [31]. The CMC/GO composite superabsorbents prepared by e-beam irradiation at 10 kGy showed a swelling capacity of 140 g g^−1^ in distilled water [32]. After analyzing the data, we concluded that the XGCMCGO hydrogels presented in this study, in addition to having the lowest concentration of 0.1% GO, had the best swelling properties in a neutral environment.

### 2.3. Diffusion of Water

The analysis of water diffusion mechanisms in swollen polymeric systems has received considerable attention in recent years due to the important applications of swollen hydrogels in biomedical and pharmaceutical engineering. When a hydrogel is in contact with water, water enters the hydrogel by diffusion and the hydrogel network expands, resulting in the swelling of the hydrogel. Diffusion involves the migration of water into pre-existing or dynamically formed spaces between the macromolecular chains of the hydrogel [33]. To follow this process, Equation (7) was applied to the initial stage of swelling process, up to 60% of maximum swelling (equilibrium state) [34]. Table 3 shows the values of water diffusion mechanisms characteristic to the XGCMCGO hydrogels.

A value of *n* ≤ 0.5 indicated a Fickian diffusion mechanism. In this case, the rate of solvent diffusion was much lower compared to the macromolecular chain relaxation of the polymer. A value of *n* in the range 0.5 < *n* < 1 indicated a non-Fickian transport, in which the diffusion of the solvent into the hydrogel structure was rapid compared to the relaxation rate of the macromolecular chain of the polymer [35].

**Figure 3:**
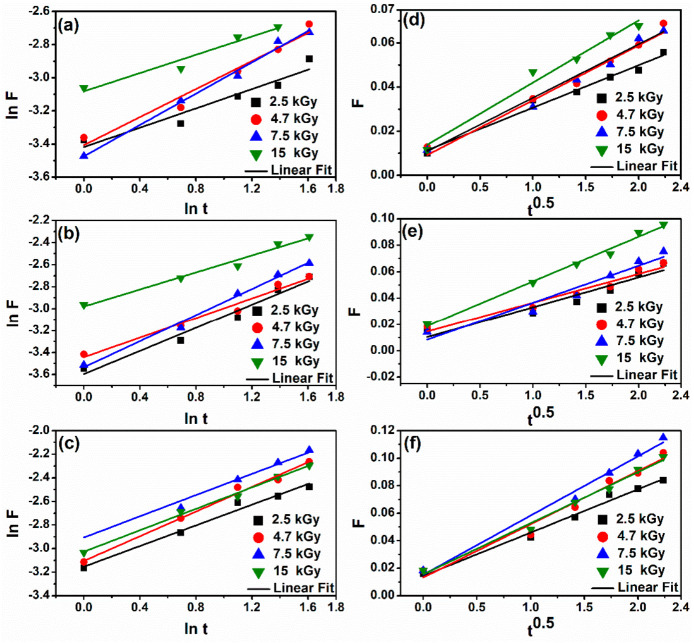
Swelling kinetics curves for the hydrogels: (**a**) XGCMCGO (50:50); (**b**) XGCMCGO (80:20); (**c**) XGCMCGO (70:30). Plots of F versus t0.5 for the hydrogels: (**d**) XGCMCGO (50:50); (**e**) XGCMCGO (80:20); (**f**) XGCMCGO (70:30).

Figure 3a–c show graphically, in logarithmic scale, the values of the parameter F = f(t). Figure 3d–f show graphically F = f (t 0.5). The values of the coefficients *n*, k, and D were calculated from the slope and intersection of the lines resulting from the swelling kinetics. The calculated values for the diffusion exponents *n* were less than 0.5 for the XGCMCGO (50:50) hydrogel at all irradiation doses, thus indicating that the solvent transport mechanism was of a Fickian type.

The XGCMCGO (80:20) hydrogel also presented a Fickian diffusion mechanism, but only in the cases of irradiation doses of 4.7 and 15 kGy. For the other irradiation doses (2.5 kGy and 7.5 kGy) the diffusion mechanism was non-Fickian. When the diffusion was non-Fickian, the relaxation time of the macromolecular chain and the diffusion had the same order of magnitude. As the solvent diffused into the hydrogel, the rearrangement of the macromolecular chains did not occur immediately.

The mechanism of water diffusion of the XGCMCGO (70:30) hydrogel was of a Fickian type; the exception was the hydrogel crosslinked with 4.7 kGy, in which case a value of *n* greater than 0.5 was obtained, thus exhibiting a non-Fickian diffusion behavior. In another similar study of a hybrid hydrogel obtained from acrylamide and XG, a value of *n* equal to 0.48 was reported [36]. Another study showed the obtaining of a GO-based hydrogel in which the inorganic component was incorporated in different concentrations. For this, values of *n* were obtained in the range of 0.5–1, which indicated a non-Fickian diffusion, which is specific to crosslinked hydrogels [37].

The values of the diffusion coefficient (D) were found in the range of 0.02–0.04 cm^2^/s, depending on the irradiation dose and the composition of the hydrogel. Compared to other studies, these values were much lower, suggesting the formation of high-molecular-weight hydrogels [38], as can be seen in the rheological measurements. For example, following a study on hydrogels obtained from CMC/acrylamide/GO by radical polymerization, the values obtained for the diffusion coefficient were in the range of 0.66–1.26 cm^2^/s [39].

### 2.4. ATR-FTIR Spectroscopy

**Figure 4:**
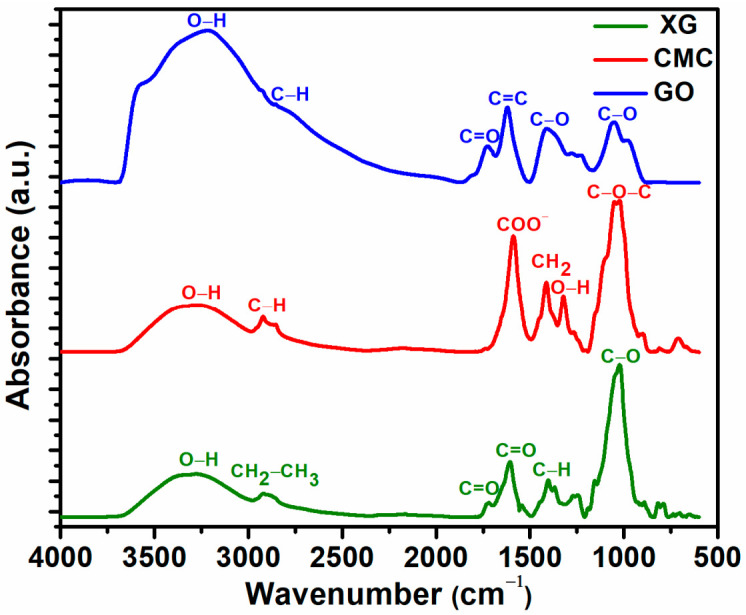
The FTIR spectra of pure XG, CMC, and pure GO.

The characteristic FTIR spectra for the natural polymers (XG and CMC) and GO are shown in Figure 4. The FTIR spectrum characteristic of XG showed: 3308 cm^−1^ (O–H groups); 2917 cm^−1^ (CH_3_ and CH_2_ functional groups); 1700–1730 cm^−1^ (C = O); 1607 cm^−1^ (C = O); 1405 cm^−1^ (C–O group); and 1025 cm^−1^ (C–O group). The FTIR spectra of CMC showed: 3303 cm^−1^ (O–H group); 2923 cm^−1^ (C–H stretching vibrations); 1586 cm^−1^ (COO– group); 1420 cm^−1^ (CH_2_); 1320 cm^−1^ (O-H groups); and 1030 cm^−1^ (C–O group). Regarding the FTIR spectrum of GO, it showed: 3231 cm^−1^ (O–H); 1732 cm^−1^ (C = O and C–H groups); 1620 cm^−1^ (C = C); 1275 cm^−1^ (C–OH); and 1053 cm^−1^ (C–O).

**Figure 5:**
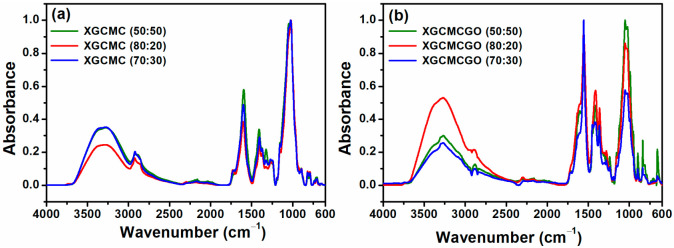
(**a**) The FTIR spectra of XGCMC without GO dried hydrogel; (**b**) the FTIR spectra of XGCMCGO dried hydrogel.

Figure 5a,b shows the characteristic FTIR spectra for nonirradiated polymeric blends with various ratios (XG:CMC) without GO (left side) and containing GO (right side). According to the acquired FTIR spectra, the displacement of the characteristic bands located in the range of 3600–600 cm^−1^ toward smaller wavenumbers corresponded to the formation of hydrogen bonds due to the miscibility of the polymers. In addition, increases in the band intensities in this area were proportional to the concentrations of natural polymers (XG and CMC) included in the polymeric blend.

For the XGCMCGO unirradiated polymeric blend, the hydrophilic functional groups (–COOH, –C = O, –OH, and –C–O–C) of GO played a key role in improving the compatibility between the polymer matrix and GO.

**Figure 6:**
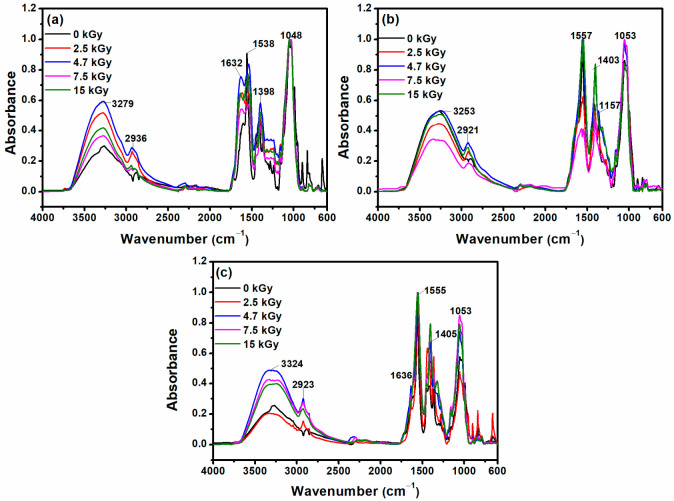
The FTIR spectra of unirradiated and irradiated dried hydrogels: (**a**) XGCMCGO (50:50); (**b**) XGCMCGO (80:20); (**c**) XGCMCGO (70:30).

The FTIR-ATR spectra, corresponding to XGCMCGO hydrogels irradiated at doses in the range of 2.5–15 kGy, are shown in Figure 6. It can be seen that the intensity of the absorption bands varied with the irradiation dose.

The intensity of specific absorption bands in the range of 3300–3000) cm^−1^ for XGCMCGO (50:50) increased with the absorbed dose. The position of the bands assigned to the groups: C–H varied from 2929 cm^−1^ to 2940 cm^−1^; C = O varied from 1614 cm^−1^ to 1637 cm^−1^; and COO– varied from 1538 cm^−1^ to 1542 cm^−1^, after e-beam irradiation at a dose of 15 kGy.

In the case of the XGCMCGO (80:20) hydrogel, the value of the band assigned to the O-H group decreased with the irradiation dose from 3268 cm^−1^ to 3253 cm^−1^. The intensity of the bands assigned to CH groups increased significantly for the irradiated polymeric blends. The decrease of the wavenumber from 1558/1412/1193/1054 cm^−1^, due to the application of a dose of 15 kGy, to 1557/1403/1157/1053 cm^−1^, respectively, may have been caused by the different degrees of crosslinking of the hydrogel. As the absorbed dose increased, the crosslinking density also increased (see Table 4).

For the XGCMCGO (70:30) hydrogels, the intensity of the absorption bands in the range of 3400–2800 cm^−1^ increased with a dose of 7.5 kGy. At this dose, the position corresponding to the OH group shifted to smaller wavenumbers, from 3333 to 3324 cm^−1^. The band assigned to the COO–group was shifted from 1556 cm^−1^ to 1550 cm^−1^ when the hydrogel was irradiated with a dose of 15 kGy. A similar trend was observed for the other bands in the range of 1500–800 cm^−1^. The intensity of the characteristic bands for the XGCMCGO (70:30) hydrogel increased when it was irradiated at doses up to 7 kGy due to the fact that the crosslinking density decreased, a direct consequence of increased swelling. These results were supported by the data obtained from swelling experiments (~6000% at a dose of 15 kGy).

### 2.5. Characterization of the Network Structure

The characterization of the network structure of a hydrogel is a complex procedure due to the many types of networks encountered in the practice; namely: regular, irregular, and weakly or strongly crosslinked networks.

Basic structural parameters for the XGCMCGO hydrogels such as the elastic modulus (G′), average molecular weight between two successive crosslinks (M_C_), crosslink density (ν_e_), and mesh size (ξ) were calculated according to the equations presented in Section 4.7. C_n_ was taken as a weighted average of the characteristic ratios of the polymers: XG = 271 [40], CMC = 10 [41] and AA = 6.7 [42]. M_r_ is the monomeric unit of XG, CMC, and AA, taken as an average (XG = 933 g/mol, CMC = 234 g/mol, and AA = 72 g/mol) [43].

Using the values of the elastic modulus (G′) determined by rheological analysis for each hydrogel composition and based on the theory of rubber elasticity, we determined M_C_. The network parameters calculated for the XGCMCGO hydrogels are presented in Table 4.

The values of the M_C_ parameter for all hydrogel compositions were in the range of 132.7–250.4 kg/mol. For the XGCMCGO (80:20) and (70:30) hydrogel compositions, the corresponding Mc values decreased with an increase in the absorbed dose; while for the XGCMCGO (50:50) hydrogel compositions, the corresponding M_C_ values increased. This increase demonstrated once again the degradation of this polymeric mixture under the action of ionizing radiation.

**Figure 7:**
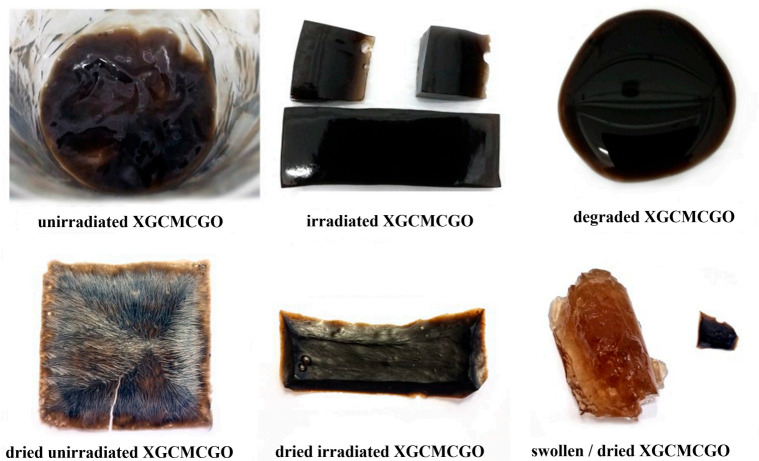
XGCMCGO hydrogels in different hypostases.

For the XGCMCGO (50:50) and (70:30) hydrogel compositions, the corresponding ξ values increased with an increase in the irradiation dose; while for the XGCMCGO (80:20) hydrogel compositions, the corresponding ξ values decreased with an increase in the irradiation dose. The decrease of the parameter ξ may have been due to the increase in the crosslinking density. Therefore, a large number of active or free radical centers were formed on the polymer chains, and a large number of small chains were formed, which led to crosslinking and the formation of a more compact network structure with a smaller mesh size. Figure 7 shows the appearance of the polymer mixture before and after irradiation with e-beams.

For this reason, ν_e_ is one of the most important structural parameters for characterizing a class of hydrogels that have superabsorbent properties. According to the values obtained, it was shown that this parameter increased as the absorbed dose increased in the cases of the XGCMCGO (80:20) and (70:30) hydrogels. The behavior was completely different in the case of XGCMCGO (50:50), where the values for ν_e_ decreased with an increase in the absorbed dose. All the values obtained for ν_e_ were in the range of 0.41–0.76 mol/m^3^, and showed the obtaining of hydrogels with a moderately crosslinked network structure that allowed the absorption of an increased amount of fluids.

Knowing the hydrogel pore size is important to understanding the mechanism for transport of bioactive substances in the macromolecular network of such materials. For example, controlling the rate of a drug diffusion is essential, because it reflects the space available for a drug molecule to diffuse inside or outside the swollen hydrogel network [44].

The values of ξ obtained for the XGCMCGO hydrogels were in the range of 123.6–210.6 nm. These values were similar to those obtained by other authors who developed hydrogels with applicability in tissue engineering, with ξ values previous reported in the range of 139–258 nm [45].

### 2.6. Rheological Analysis

The hydrogels were evaluated to determine the viscoelastic parameters G′ and G″. The elastic modulus (G′) shows the elasticity of the crosslinked bonds and the total elasticity of the material, while the loss or viscous modulus (G″) provides a perspective on the viscosity of the material. The ratio between G′ and G″ indicates the total resistance to deformation of the material [46].

The mechanical properties of hydrogels are important when selecting material for biomedical and other applications. They depend on the composition of the hydrogel and its water content. Hydrogels with a higher water content generally have a better permeability and biocompatibility [47]. However, there are some disadvantages, as a high degree of swelling is accompanied by a decrease in mechanical strength. For many applications, the combination of a high swelling degree and good mechanical properties is very important. Many approaches have been used to improve the mechanical properties of hydrogels, including the copolymerization of hydrophilic and hydrophobic monomers, increasing the crosslinking density, and varying the polymerization conditions [48].

**Figure 8:**
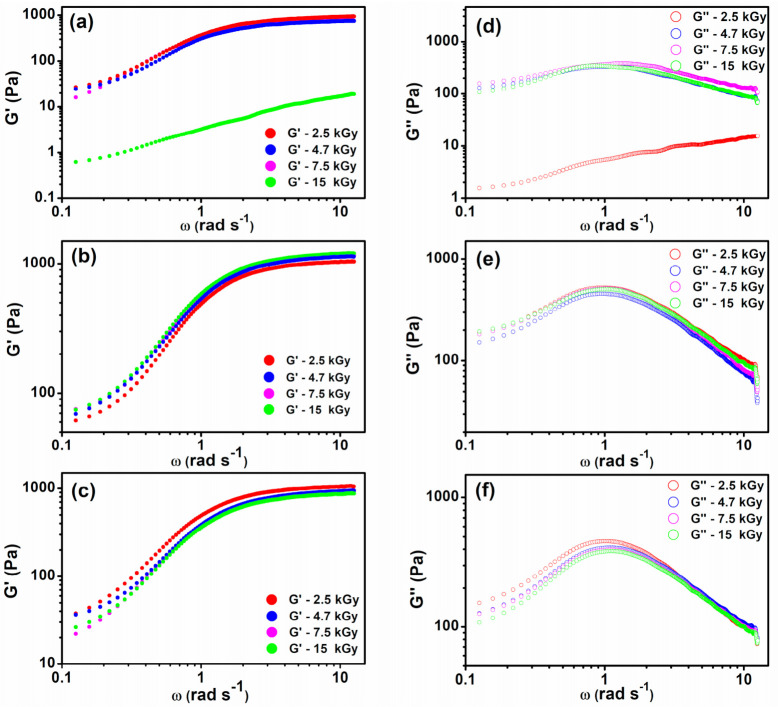
Elastic modulus (G′), as a function of the angular frequency (ω), of the (**a**) XGCMCGO (50:50), (**b**) XGCMCGO (80:20), and (**c**) XGCMCGO (70:30) hydrogels and their evolutions as a function of the absorbed dose. Viscous modulus (G″), as a function of the angular frequency (ω), of the (**d**) XGCMCGO (50:50), (**e**) XGCMCGO (80:20), and (**f**) XGCMCGO (70:30) hydrogels, and their evolutions as a function of the absorbed dose.

Figure 8 shows the variation of the elastic and viscous (G′ and G″) moduli as a function of angular frequency (ω) and the absorbed dose for the XGCMCGO hydrogels. We observed that G′ was greater than G″ for all the hydrogel compositions. This is a critical requirement for hydrogels, as a G′ greater than the G″ suggests that the hydrogel has a higher elastic behavior [49].

Rheological analysis confirms the crosslinking or degradation processes that occur depending on the composition of the polymer mixture. Moreover, the effect of irradiation at various irradiation doses is highlighted, and the G′ and G″ is dependent on the composition of the hydrogel and the absorbed dose. A rheological analysis was performed on swollen hydrogel samples. The determined values of G′ and G″ are shown in Table 4.

For the XGCMCGO (80:20) hydrogel, the G′ increased with an increase in the absorbed dose, with a maximum value for G′ = 1052 Pa at 15 kGy. The XGCMCGO (50:50) hydrogel had a G′ = 869 Pa at 2.5 kGy. When this polymeric blend was irradiated with 15 kGy, the G′ decreased very drastically down to 12 Pa. In the case of the XGCMCGO (70:30) hydrogel, G′ = 913 Pa was obtained at 2.5 kGy, and at 15 kGy, G′ = 766 Pa.

The G′ decreased with the absorbed dose in the cases of the XGCMCGO (50:50) and (70:30) hydrogels. We concluded that the decrease in G′ reflected the reduction of the crosslinking density (ν_e_), while the decrease in G″ was due to the inhibition of the viscous behavior, showing a complete degradation of the polymer blend.

The XGCMCGO (80:20) hydrogel had a very well defined elastic behavior depending on the absorbed dose. In a recent study, a superabsorbent hydrogel based on CMC/starch/GO presented a value of G′ = 8050 Pa [50]. In another study, hydrogels obtained from CMC by redox polymerization for soft-tissue healing applications had a value of G′ = 814 Pa [45]. As shown by the rheological analysis and the sol-gel analysis, the XGCMCGO (80:20) hydrogel had the best properties.

## 3. Conclusions

We prepared novel and complex XGCMCGO hydrogels by e-beam crosslinking to be used as a potential substrate in biomedical engineering. Irradiation of XGCMC polymeric blends with e-beams proved to be a suitable technique, especially when GO was incorporated into their composition. Irradiation of polymeric mixtures with a wide range of doses allowed us to obtain hydrogels with different properties.

XGCMCGO hybrid hydrogels had better mechanical strength compared to the pure CMC hydrogel, and a better flexibility and improved swelling behavior than the simple XG hydrogel.

The gel fraction and the swelling properties of the prepared hydrogels depended on the composition of the polymers and the absorbed dose.

The hydrogels showed superabsorbent capacity and reached equilibrium in less than 6 h, especially the XGCMCGO (70:30) hydrogel, which reached the highest swelling degree of about 6000%, at 15 kGy. The crosslinking process predominated compared to the degradation process.

By characterizing the network structure, we observed that the hybrid hydrogels with high XG concentration showed the best structural properties. The crosslinking density increased with the absorbed dose.

The mesh size obtained from the experimental data of rheological analysis was in the range of 123–210 nm; these values were comparable to those of several hydrogels.

The interaction between the hydrogel components was highlighted by FT-IR analysis, and the increase in absorption band intensities for the characteristic functional group, as well as their shifting toward lower wavenumbers, were correlated with the crosslinking degree, which decreased at a dose of 15 kGy.

For all hydrogel compositions, G′ > G″, thus suggesting that the hydrogels had a higher elastic behavior. The G′ had values in a wide range of 12–1052 Pa, with the maximum value obtained at a dose of 15 kGy for the XGCMCGO (80:20) hydrogel composition.

Therefore, the prepared XGCMCGO superabsorbent hydrogels belong to a class of environmentally friendly materials, and might have potential practical applications in many areas, such as in biomedical engineering and hygienic products.

## 4. Materials and Methods

### 4.1. Materials

Sodium carboxymethylcellulose (CMC, Mw = 2.5 × 10^5^ g/mol), *N′N*-methylene-bis-acrylamide (NMBA 99%, Mw = 154.17 g/mol), acrylic acid anhydrous 99% containing MEHQ as inhibitor (AA, Mw = 72.06 g/mol), and NaOH were purchased from Merck KGaA, Darmstadt, Germany. A commercial xanthan gum (XG—food grade, produced by Jungbunzlauer, Wien, Austria) in powder form with a molecular weight of Mw = 1.6 × 10^6^ g/mol was used. Ultra-highly concentrated single-layer graphene oxide (6.2 g/L) was purchased from Graphene Laboratory Inc., New York, NY, USA.

### 4.2. Synthesis of Hydrogels and E-Beam Irradiation

In this experiment, three different hydrogels based on XGCMCGO with different content of XG and CMC in the presence of AA, NaOH, and NMBA were prepared. XG (5 wt %) and CMC (2 wt %) were dissolved in DI water at room temperature. After complete solubilization of the XG and CMC, they were mixed with each other in different compositions. For each XG:CMC ratio, 75 mL of the mixture was prepared. The ratios of XG:CMC in the mixtures were 50:50; 80:20, and 70:30. For a better understanding of the experiments, sample compositions are presented in Table 5.

Then, 15 mL of each homogeneous solution of XGCMCGO was packed in a hermetically sealed polyethylene zip bag and subjected to e-beam irradiation at predetermined doses (2.5–15 kGy). The e-beam sample irradiation was performed in air at room temperature (25 °C) using a linear electron accelerator (National Institute for Laser, Plasma and Radiation Physics, Măgurele, Romania) at a fixed beam energy of 6 MeV (average beam current of 10 μA, pulse length of 3.75 μs, pulse repetition rate of 50 Hz, and average dose rate of 1 kGy/min) [51]. The dosimetry was performed using graphite calorimeters.

### 4.3. Sol-Gel Analysis

The hydrogel samples were dried in a vacuum oven to a constant weight and then immersed in DI water for 48 h at room temperature (25 °C). After 48 h, the swollen hydrogels were removed from the water and dried at 30 °C to a constant weight. The gel fraction (GF) and soluble fraction (s) were calculated as follows:

(1) $$G\left(\%\right)\;=\;\left(\frac{W_{d}}{W_{i}}\right)$$

(2) s=1−G

where W_i_ is the initial weight of dried sample after irradiation, W_d_ is the weight of the dried insoluble part of sample after immersion for 48 h, and s is soluble fraction of the polymer. All measurements were carried out in triplicate for each sample, and all values were expressed as mean value and standard deviation of three independent samples.

The gelation doses (the doses necessary to produce the first insoluble gel fraction) and the degradation vs. crosslinking ratios for the XGCMCGO hydrogels were calculated using a customized computer program for sol-gel analysis (Gelsol95), which was based on the Charlesby–Rosiak formula [26]:

(3) $$s+\sqrt{s}=\frac{p_{0}}{q_{0}}+\left(2-\frac{p_{0}}{q_{0}}\right)\left(\frac{D_{v}+D_{g}}{D_{v}+D}\right)$$

where *p*_0_ is the degradation density (i.e., average number of main chain scissions per monomer unit and per unit dose), *q*_0_ is the crosslinking density (i.e., fraction of monomer units crosslinked per unit dose), D is the absorbed dose (kGy), D_g_ is the gelation dose (kGy), and D_V_ is the virtual dose (kGy) (the dose necessary to transform the real sample into a sample with the molecular weight distribution of M_w_/M_n_ = 2).

The radiation yields of crosslinking and degradation (scission) were calculated using the following equations:

(4) $$G\left(X\right)\;=\frac{4.9\cdot 10^{2}\cdot c}{M_{C}\cdot D\cdot \rho }$$

(5) $$G\left(S\right)\;=G\left(X\right)\cdot 2\frac{p_{0}}{q_{0}}$$

where G(X) is the radiation yield of crosslinking (expressed as number of moles of crosslinking bonds per Joule), G(S) is the radiation yield of chain scission (mol/J), M_C_ (kg/mol) is the average molecular weight between two successive crosslinks, c (g/L) is the polymer concentration in irradiated solution, D is absorbed dose (J/kg), and ρ (kg/m^3^) is the polymer density [52].

### 4.4. Swelling Degree

The swelling properties of hydrogels were explored by placing the dried hydrogels in DI water at room temperature for 48 h to reach swelling equilibrium. At specified times, the swelled hydrogels were taken out of the distilled water, blotted with paper, weighed, and immersed again.

The swelling degree (SD(%)) was calculated as a function of the dry (W_d_) and swollen (W_s_) hydrogel weights using Equation (6) [53]:

(6) $$\mathrm{SD}\left(\%\right)\;=\frac{\left(W_{s}-W_{d}\right)}{W_{d}}\cdot 100$$

### 4.5. Diffussion of Water

The most basic law of Fick’s was used for the explanation of swelling kinetics and diffusion of the polymeric structures. The following equation was used to determine the nature of diffusion of water into hydrogels [54]:

(7) $$F=\frac{M_{t}}{M_{\infty }}={\mathrm{kt}}^{n}$$

where F is the fraction of swelling due to the water uptake, M_t_ is the adsorbed water at time t, M_∞_ is the adsorbed water at equilibrium, k is a proportionality constant, and *n* is the diffusional exponent. The first 60% of the water uptake data were fitted to Equation (7), and the corresponding values of k and n were obtained.

### 4.6. ATR-FTIR Spectroscopy

The changes in chemical structure of the crosslinked hydrogels were investigated. ATR-FTIR spectra of unirradiated and irradiated samples were taken with a PerkinElmer Spectrum 100 FTIR Spectrometer. The samples for FTIR analysis were first dried in a vacuum oven at 30 °C for 72 h. The samples were subjected to wavenumbers ranging from 4000 to 600 cm^−1^ at ambient temperature and a resolution of 4 cm^−1^, averaged from 50 scans/sample.

### 4.7. Characterization of Network Structure

Network parameters of the XGCMCGO hydrogels, such as the average molecular weight between two crosslinks (Mc), crosslinking density (νe), and mesh size (ξ), were determined by using the swelling and rheological measurements. Using elastic modulus (G′) values determined from rheological measurements and based on the rubber elasticity theory, Mc could be determined using the following equation [55]:

(8) $$M_{C}=\frac{A\rho \mathrm{RT}{\left(\nu_{2r}\right)}^{2/3}{\left(\nu_{2s}\right)}^{1/3}}{G^{\prime }}$$

where R is the universal gas constant (8.314 m^3^ Pa/molK), T is the absolute experimental temperature (298.15 °K), ν_2r_ is the polymer volume fraction after e-beam crosslinking, ν_2s_ is the polymer volume fraction of the crosslinked hydrogel in swollen state, ρ (kg/m^3^) is the polymer density, and the factor A equals 1 for an affine network and 1–2/ϕ for a phantom network.

The effective crosslink density (ν_e_) of the hydrogels was calculated using Equation (9):

(9) $$\nu_{e}=\frac{\rho }{M_{C}}$$

The polymer volume fractions (ν_2r_ and ν_2s_) were determined using Equation (10):

(10) $$\nu_{2r\left(s\right)}=\frac{{\left[1+\left(w_{2r\left(s\right)}-1\right)\cdot \rho_{\mathrm{hydrogel}}\right]}^{-1}}{\rho_{\mathrm{solvent}}}$$

where ρ_hydrogel_ and ρ_solvent_ are the densities of the hydrogel and solvent (kg/m^3^), respectively; and *w*_2r(s)_ is the weight of the hydrogel after e-beam crosslinking after swelling (g). The weight swelling ratio of hydrogels after crosslinking (*w*_2__r_) was calculated as: *w*_2__r_ = hydrogel mass after irradiation/hydrogel dry mass. The weight swelling ratio of hydrogels after swelling (*w*_2s_) was calculated as: *w*_2s_ = hydrogel mass after swelling/hydrogel dry mass.

The mesh size of the polymer network (ξ) was determined using Equation (11) [56]:

(11) ξ=ν2s−1/3·Cn2MCMr−12·l

where C_n_ is the Flory characteristic ratio, M_r_ is the average molecular weight of the repeating unit, and l is the carbon–carbon bond length (0.154 nm).

### 4.8. Dynamic Rheological Measurements

Dynamic rheological measurements of the hydrogels were performed by employing an MFR 2100 Micro Fourier Rheometer (GBC, Australia) equipped with a home-made temperature control jacket connected to a Lauda E100 circulating water bath.

The operating parameters of the instrument during rheological investigation were as follows: gap between plates—400 μm; displacement amplitude—0.03 μm (to fall into the linear viscoelasticity domain); frequency domain—0.005–2.000 Hz (with a step of 0.005 Hz, which led to angular frequencies, in rad/s, of 2π times higher than the corresponding frequencies taken in Hz); equilibration time for each of the isothermal measurements—20 min; and 30 scans per rheogram.

The dynamic rheological parameters of storage modulus (G′) and loss modulus (G″) were determined to evaluate the stability of the hydrogel network. All rheological measurements were performed in triplicate at the same constant temperature of 23 °C, and all values were expressed as mean value and standard deviation.

## Figures and Tables

**Table 1:** Sol-gel parameters calculated according the Charlesby–Rosiak Equation (3).

Hydrogel	*p*_0_/*q*_0_	Dg (kGy)
XGCMCGO (50:50)	1.04	0.23
XGCMCGO (80:20)	0.64	0.24
XGCMCGO (70:30)	0.66	0.79

**Table 2:** Radiation-chemical yield of crosslinking G(X) and chain scission G(S) calculated for the XGCMCGO hydrogels.

Dose (kGy)	Radiation-Chemical Yields (μmol/J)	XGCMCGO (50:50)	XGCMCGO (80:20)	XGCMCGO (70:30)
2.5	G(X)	121.4	113.7	132.2
	G(S)	252.5	100.1	121.6
4.7	G(X)	54.4	65.8	73.5
	G(S)	113.2	57.9	67.6
7.5	G(X)	33.6	46.8	53.4
	G(S)	69.8	41.2	49.1
15	G(X)	-	27.6	27.9
	G(S)	-	24.3	25.6

**Table 3:** Parameters k, *n*, and D for the XGCMCGO hydrogels.

**XGCMCGO (50:50)**						
Dose (kGy)	k	*n*	R^2^	D (cm^2^/s)	R^2^	Mechanism
2.5	−3.42	0.29	0.91	0.02	0.98	Fickian
4.7	−3.41	0.42	0.96	0.03	0.98	Fickian
7.5	−3.47	0.47	0.99	0.03	0.98	Fickian
15	−3.08	0.28	0.94	0.03	0.98	Fickian
**XGCMCGO (80:20)**						
Dose (kGy)	k	*n*	R^2^	D (cm^2^/s)	R^2^	Mechanism
2.5	−3.59	0.52	0.96	0.02	0.92	Non—Fickian
4.7	−3.44	0.45	0.97	0.03	0.95	Fickian
7.5	−3.53	0.59	0.99	0.03	0.93	Non—Fickian
15	−2.98	0.38	0.97	0.04	0.99	Fickian
**XGCMCGO (70:30)**						
Dose (kGy)	k	*n*	R^2^	D (cm^2^/s)	R^2^	Mechanism
2.5	−3.15	0.44	0.98	0.03	0.98	Fickian
4.7	−2.94	0.52	0.99	0.04	0.97	Non—Fickian
7.5	−2.93	0.45	0.98	0.04	0.99	Fickian
15	−2.94	0.46	0.99	0.03	0.99	Fickian

**Table 4:** The elastic modulus (G′), the molecular weight between two crosslinks of the hydrogel chains (M_C_), the crosslink density (ν_e_), and network mesh size (ξ) of the XGCMCGO hydrogels.

**XGCMCGO (50:50)**				
Dose (kGy)	G′ (Pa)	M_C_ (kg/mol)	ν_e_ (mol/m^3^)	ξ (nm)
2.5	869	132.7	0.76	140.1
4.7	712	157.3	0.64	158.4
7.5	646	160.3	0.63	170.6
15	200	-	-	-
**XGCMCGO (80:20)**				
Dose (kGy)	G′ (Pa)	M_C_ (kg/mol)	ν_e_ (mol/m3)	ξ (nm)
2.5	912	210.7	0.49	138.5
4.7	933	195.9	0.52	132.8
7.5	1020	172.1	0.59	128.8
15	1052	146.5	0.70	123.6
**XGCMCGO (70:30)**				
Dose (kGy)	G′ (Pa)	M_C_ (kg/mol)	ν_e_ (mol/m3)	ξ (nm)
2.5	1016	250.4	0.41	145.9
4.7	840	241.3	0.43	171.8
7.5	795	207.8	0.49	191.7
15	766	200.1	0.52	210.6

**Table 5:** Sample composition details.

Hydrogel	Chemical Composition (%, *w*/*v*)					
	XG	CMC	GO	AA	NaOH	NMBA
XGCMCGO (50:50)	2.1	0.8	0.1	0.8	0.4	2
XGCMCGO (80:20)	3.3	0.3	0.1	2.8	1.4	1.5
XGCMCGO (70:30)	2.9	0.5	0.1	5.9	2.9	0.5

## References

[1] Qiu Y., Park K. (2001). Environment-sensitive hydrogels for drug delivery. Adv. Drug Deliv. Rev..

[2] Miyata T., Uragami T., Nakamae K. (2002). Biomolecule-sensitive hydrogels. Adv. Drug Deliv. Rev..

[3] Gupta P., Vermani K., Garg S. (2002). Hydrogels: From controlled release to pH-responsive drug delivery. Drug Discov. Today.

[4] Guo S., Dong S. (2011). Graphene nanosheet: Synthesis, molecular engineering, thin film, hybrids, and energy and analytical applications. Chem. Soc. Rev..

[5] Chen Y., Chen L., Bai H., Li L. (2013). Graphene oxide–chitosan composite hydrogels as broad-spectrum adsorbents for water purification. J. Mater. Chem. A.

[6] Gao H., Sun Y., Zhou J., Xu R., Duan H. (2013). Mussel-Inspired Synthesis of Polydopamine-Functionalized Graphene Hydrogel as Reusable Adsorbents for Water Purification. ACS Appl. Mater. Interfaces.

[7] Wang J., Su S., Qiu J. (2017). Biocompatible swelling graphene oxide reinforced double network hydrogels with high toughness and stiffness. New J. Chem..

[8] Peng S., Han X., Li L., Zhu Z., Cheng F., Srinivansan M., Adams S., Ramakrishna S. (2016). Unique Cobalt Sulfide/Reduced Graphene Oxide Composite as an Anode for Sodium-Ion Batteries with Superior Rate Capability and Long Cycling Stability. Small.

[9] Xu H., Adolfsson K., Xie L., Hassanzadeh S., Pettersson T., Hakkarainen M. (2016). Zero-Dimensional and Highly Oxygenated Graphene Oxide for Multifunctional Poly(lactic acid) Bionanocomposites. ACS Sustain. Chem. Eng..

[10] Huang Y., Zeng M., Ren J., Wang J., Fan L., Xu Q. (2012). Preparation and swelling properties of graphene oxide/poly(acrylic acid-co-acrylamide) super-absorbent hydrogel nanocomposites. Colloids Surf. A Physicochem. Eng. Asp..

[11] Lei H., Xie M., Zhao Y., Zhang F., Xu Y., Xie J. (2016). Chitosan/sodium alginate modificated graphene oxide-based nanocomposite as a carrier for drug delivery. Ceram. Int..

[12] Geim A.K., Novoselov K.S. (2007). The rise of graphene. Nat. Mater..

[13] Balandin A.A., Ghosh S., Bao W., Calizo I., Teweldebrhan D., Miao F., Lau C.N. (2008). Superior Thermal Conductivity of Single-Layer Graphene. Nano Lett..

[14] Lee C., Wei X., Kysar J., Hone J. (2008). Measurement of the Elastic Properties and Intrinsic Strength of Monolayer Graphene. Science.

[15] Li Y., Ha Y., Wang F., Li Y. (2011). Effect of Irradiation on the Molecular Weight, Structure and Apparent Viscosity of Xanthan Gum in Aqueous Solution. Adv. Mater. Res..

[16] Sen M., Hayrabolulu H., Taskin P., Torun M., Cutrubinis M., Güven O. (2015). Radiation induced degradation of xanthan gum in the solid state. Radiat. Phys. Chem..

[17] Hayrabolulu H., Cutrubinis M., Güven O., Sen M. (2017). Radiation induced degradation of xanthan gum in aqueous solution. Radiat. Phys. Chem..

[18] Hayrabolulu H., Demeter M., Cutrubinis M., Şen M. (2021). Radiation synthesis and characterization of xanthan gum hydrogels. Radiat. Phys. Chem..

[19] Choi J., Lee H.-S., Kim J.-H., Lee K.W., Lee J., Seo S.J., Kang K.W., Byun M.W. (2008). Controlling the radiation degradation of carboxymethylcellulose solution. Polym. Degrad. Stab..

[20] Sung Y., Kim T.-H., Lee B. (2016). Syntheses of carboxymethylcellulose/graphene nanocomposite superabsorbent hydrogels with improved gel properties using electron beam radiation. Macromol. Res..

[21] Lee S., Lee H., Sim J.H., Sohn D. (2014). Graphene oxide/poly(acrylic acid) hydrogel by γ-ray pre-irradiation on graphene oxide surface. Macromol. Res..

[22] Pinelli F., Nespoli T., Rossi F. (2021). Graphene Oxide-Chitosan Aerogels: Synthesis, Characterization, and Use as Adsorbent Material for Water Contaminants. Gels.

[23] Calina I., Demeter M., Vancea C., Scarisoreanu A., Meltzer V. (2018). E-beam radiation synthesis of xanthan-gum/carboxymethylcellulose superabsorbent hydrogels with incorporated graphene oxide. J. Macromol. Sci. Part A.

[24] Das B., Prasad K.E., Ramamurty U., Rao C. (2009). Nano-indentation studies on polymer matrix composites reinforced by few-layer graphene. Nanotechnology.

[25] Said H.M., Alla S.G.A., El-Naggar A.W.M. (2004). Synthesis and characterization of novel gels based on carboxymethyl cellulose/acrylic acid prepared by electron beam irradiation. React. Funct. Polym..

[26] Olejniczak J., Rosiak J., Charlesby A. (1991). Gel/dose curves for polymers undergoing simultaneous crosslinking and scission. Int. J. Radiat. Appl. Instrum. Part C. Radiat. Phys. Chem..

[27] Mozalewska W., Czechowska-Biskup R., Olejnik A.K., Wach R.A., Ulański P., Rosiak J.M. (2017). Chitosan-containing hydrogel wound dressings prepared by radiation technique. Radiat. Phys. Chem..

[28] Salmawi K., El-Naggar A., Ibrahim S. (2016). Gamma Irradiation Synthesis of Carboxymethyl Cellulose/Acrylic Acid/Clay Superabsorbent Hydrogel. Adv. Polym. Technol..

[29] Sultana S., Islam M.R., Dafader N.C., Haque M.E. (2012). Preparation of carboxymethyl cellulose/acrylamide copoly-mer hydrogel using gamma radiation and investigation of its swelling behavior. J. Bangladesh Chem. Soc..

[30] Hosseini S.M., Shahrousvand M., Shojaei S., Khonakdar H.A., Asefnejad A., Goodarzi V. (2020). Preparation of superabsorbent eco-friendly semi-interpenetrating network based on cross-linked poly acrylic acid/xanthan gum/graphene oxide (PAA/XG/GO): Characterization and dye removal ability. Int. J. Biol. Macromol..

[31] Wang Z., Ning A., Xie P., Gao G., Xie L., Li X., Song A. (2017). Synthesis and swelling behaviors of carboxymethyl cellulose-based superabsorbent resin hybridized with graphene oxide. Carbohydr. Polym..

[32] Kim B., Kim T.-H., Lee B. (2021). Optimal synthesis of carboxymethylcellulose-based composite superabsorbents. Korean J. Chem. Eng..

[33] Saraydın D., Isikver Y., Sahiner N., Güven O. (2004). The Influence of Preparation Methods on the Swelling and Network Properties of Acrylamide Hydrogels with Crosslinkers. J. Macromol. Sci. Part A.

[34] Karadağ E., Saraydın D., Güven O. (1997). Influence of some crosslinkers on the swelling of acrylamide-crotonic acid hydrogels. Turkish J. Chem..

[35] Colombo P., Bettini R., Santi P., Peppas N.A. (2000). Swellable matrices for controlled drug delivery: Gel-layer behaviour, mechanisms and optimal performance. Pharm. Sci. Technolo. Today.

[36] Karadağ E., Ödemiş H., Kundakçi S., Üzüm Ö.B. (2016). Swelling characterization of acrylamide/zinc acrylate/xanthan gum/sepiolite hybrid hydrogels and Its application in sorption of janus green B from aqueous solutions. Adv. Polym. Technol..

[37] Dai H., Zhang Y., Ma L., Zhang H., Huang H. (2019). Synthesis and response of pineapple peel carboxymethyl cellulose-g-poly (acrylic acid-co-acrylamide)/graphene oxide hydrogels. Carbohydr. Polym..

[38] Siouffi A., Guiochon G. (2000). Chromatography: Thin-Layer (PLANAR)|Theory of Thin-Layer (Planar) Chromatography. Encycl. Sep. Sci..

[39] Varaprasad K., Jayaramudu T., Sadiku E.R. (2017). Removal of dye by carboxymethyl cellulose, acrylamide and graphene oxide via a free radical polymerization process. Carbohydr. Polym..

[40] Borsali R., Pecora R. (2008). Soft-Matter Characterization.

[41] Robinson G., Ross-Murphy S.B., Morris E.R. (1982). Viscosity-molecular weight relationships, intrinsic chain flexibility, and dynamic solution properties of guar galactomannan. Carbohydr. Res..

[42] Gudeman L.F., Peppas N.A. (1995). pH-sensitive membranes from poly (vinyl alcohol)/poly (acrylic acid) interpenetrating networks. J. Membr. Sci..

[43] Fekete T., Borsa J., Takács E., Wojnarovits L. (2015). Synthesis of carboxymethylcellulose/acrylic acid hydrogels with superabsorbent properties by radiation-initiated crosslinking. Radiat. Phys. Chem..

[44] Wong R., Ashton M., Dodou K. (2015). Effect of Crosslinking Agent Concentration on the Properties of Unmedicated Hydrogels. Pharmaceutics.

[45] Varma D.M., Gold G.T., Taub P.J., Nicoll S.B. (2014). Injectable carboxymethylcellulose hydrogels for soft tissue filler applications. Acta Biomater..

[46] Kalia S., Choudhury A.R. (2019). Synthesis and rheological studies of a novel composite hydrogel of xanthan, gellan and pullulan. Int. J. Biol. Macromol..

[47] Micic M., Suljovrujic E. (2013). Network parameters and biocompatibility of p(2-hydroxyethyl methacrylate/itaconic acid/oligo(ethylene glycol) acrylate) dual-responsive hydrogels. Eur. Polym. J..

[48] Lorenzo E., Katime I. (2003). Some Mechanical Properties of Poly[(acrylic acid)-co-(itaconic acid)] Hydrogels. Macromol. Mater. Eng..

[49] Lejardi A., Hernandez R., Gonzalez M.C., Santos J., Etxeberria A., Sarasua J.-R., Mijangos C. (2014). Novel hydrogels of chitosan and poly(vinyl alcohol)-g-glycolic acid copolymer with enhanced rheological properties. Carbohydr. Polym..

[50] Braihi A. (2015). Viscoelastic and Rheological Properties of Carboxymethyl Cellulose/Starch/Graphite Oxide as Superabsorbent Hydrogel Nano Composites (SHNCs). Int. J. Mater. Sci. Appl..

[51] Manaila E., Craciun G., Ighigeanu D., Lungu B., Dumitru M., Stelescu M. (2021). Electron Beam Irradiation: A Method for Degradation of Composites Based on Natural Rubber and Plasticized Starch. Polymers.

[52] Suhartini M., Yoshii F., Nagasawa N., Mitomo H. (2011). Radiation Yield and Radicals Produced in Irradiated Poly (Butylene Succinate). Atom Indon..

[53] Nagasawa N., Yagi T., Kume T., Yoshii F. (2004). Radiation crosslinking of carboxymethyl starch. Carbohydr. Polym..

[54] Ritger P.L., Peppas N.A. (1987). A simple equation for description of solute release I. Fickian and non-fickian release from non-swellable devices in the form of slabs, spheres, cylinders or discs. J. Control. Release.

[55] Mahmudi N., Sen M., Rendevski S., Güven O. (2007). Radiation synthesis of low swelling acrylamide based hydrogels and determination of average molecular weight between cross-links. Nucl. Instrum. Methods Phys. Res. Sect. B Beam Interact. Mater. Atoms.

[56] Canal T., Peppas N.A. (1989). Correlation between mesh size and equilibrium degree of swelling of polymeric networks. J. Biomed. Mater. Res..

